# A Simple FCM Method to Avoid Misinterpretation in *Saccharomyces cerevisiae* Cell Cycle Assessment between G0 and Sub-G1

**DOI:** 10.1371/journal.pone.0084645

**Published:** 2014-01-02

**Authors:** Pierre Delobel, Catherine Tesnière

**Affiliations:** 1 INRA, UMR1083, Montpellier, France; 2 SupAgro Montpellier, UMR1083, Montpellier, France; 3 Universit Montpellier 1, UMR1083, Montpellier, France; Texas A&M University, United States of America

## Abstract

Extensively developed for medical and clinical applications, flow cytometry is now being used for diverse applications in food microbiology. Most uses of flow cytometry for yeast cells are derived from methods for mammalian cells, but yeast cells can present specificities that must be taken into account for rigorous analysis of the data output to avoid any misinterpretation. We report an analysis of *Saccharomyces cerevisiae* cell cycle progression that highlights possible errors. The cell cycle was analyzed using an intercalating fluorochrome to assess cell DNA content. In analyses of yeast cultures, the presence of a sub-G1 peak in the fluorescent signal is often interpreted as a loss of DNA due to its fragmentation associated with apoptosis. However, the cell wall and its stucture may interfere with the fluorescent signal recorded. These observations indicate that misinterpretation of yeast DNA profiles is possible in analyses based on some of the most common probes: cells in G0 appeared to have a lower DNA content and may have been mistaken as a sub-G1 population. However, careful selection of the fluorochrome for DNA quantification allowed a direct discrimination between G0 and G1 yeast cell cycle steps, without additional labeling. We present and discuss results obtained with five current fluorochromes. These observations led us to recommend to use SYTOX Green for cycle analysis of living cells and SYBR Green I for the identification of the apoptosis sub-G1 population identification or the DNA ploidy application.

## Introduction

**Figure 1:**
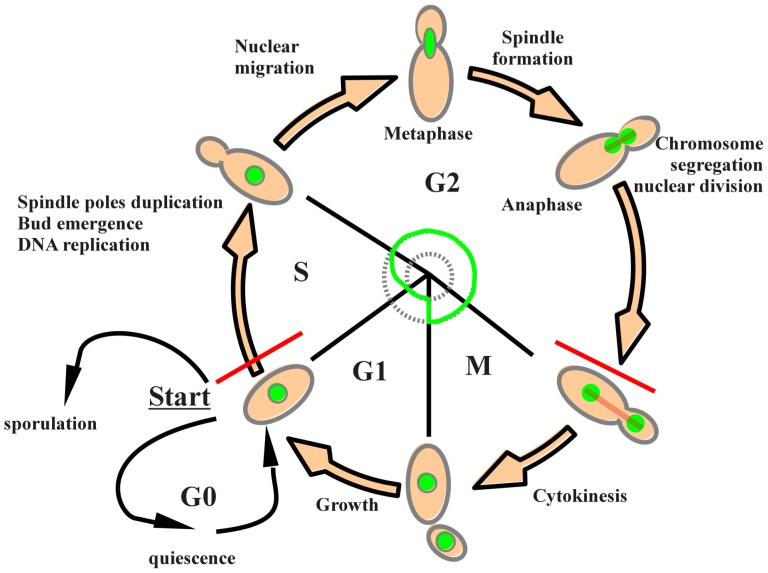
Budding yeast cell progression through the cell cycle.

The yeast *Saccharomyces cerevisiae* has been used as a model system for the study of the eukaryotic cell cycle [1]. Its main experimental advantage is the ease with which the cell cycle can be analyzed by simple culture techniques. In a proliferating population, the distribution of cells within the major distinct phases of the cell cycle (Figure 1) can be determined from differences in DNA content between cells in prereplicative phases (G0 and G1), cells replicating DNA (S), and post-replicative plus mitotic (G2+M) phase cells. Cells in G2 and M phases have identical DNA content and thus cannot be discriminated on this basis. Cells with fractional DNA content, a situation occuring during apoptosis, can be identified as a “sub-G1” population [2]. The progression of the cycle in yeast cells can be investigated by flow cytometry (FCMFCMFlow cytometry) following fixation and staining with a fluorescent dye that binds stoichiometrically to DNA. FCM is a powerful and widely used approach to estimating cell cycle phase distribution and analyzing many aspects of cell cycle regulation in diverse cell types [3]. However, some of the specificities of yeast need be taken into account when using this technique. For instance, the percentage of each yeast population represents the time spent in each phase: in a rich medium during exponential growth G1 phase represents only 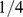 of the total cycle, whereas the S-G2/M phases last about 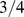 of the cell cycle [4]. In addition, doublets and gating difficulties can be encountered in this budding yeast population when analyzed by FCM.

Fluorochromes have different characteristics that can influence the evaluation of DNA content and help to improve assessments of the yeast cell cycle. For instance, some fluorochromes are DNA specific or not, GC selective or general intercalating dyes. Thus, we tested and compared five current fluorochromes (SYTOX Green, propidium iodide [PI]PIpropidium iodide, TO-PRO-3, 7-aminoactinomycin D [7-AAD]7-AAD7-aminoactinomycin D and SYBR Green I) that could resolve DNA quantification and/or cell cycle status. For that, a single cell preparation was performed, including RNase treatment. Then, the preparation was labeled by each fluorochrome individually.

We report a critical analysis of evaluations of the budding yeast cell cycle by FCM with single DNA staining, and their interpretation. We indicate how the problems identified can be solved.

## Results

### Growth Kinetics

Cytometry measurements giving aberrant data concerning the presence of sub-G1 populations in *S. cerevisiae* samples (data not shown) led us to analyze the method for studying this yeast cell cycle. In particular, we looked at the choice of the fluorochrome using standard cultures.

**Figure 2:**
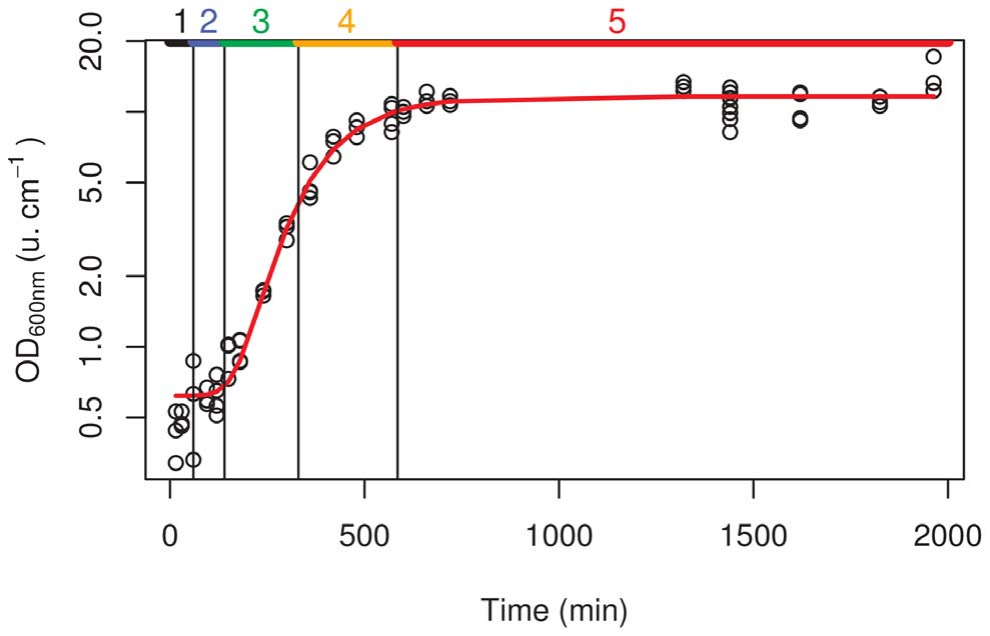
Evolution of cell density and identification of growth stages before DNA staining. Five growth stages were defined according to log OD of the cell culture: 1: lag phase (black), 2: start of growth (cyan), 3: exponential stage (green), 4: slowdown phase (orange), 5: stationary phase (red).

**Figure 3:**
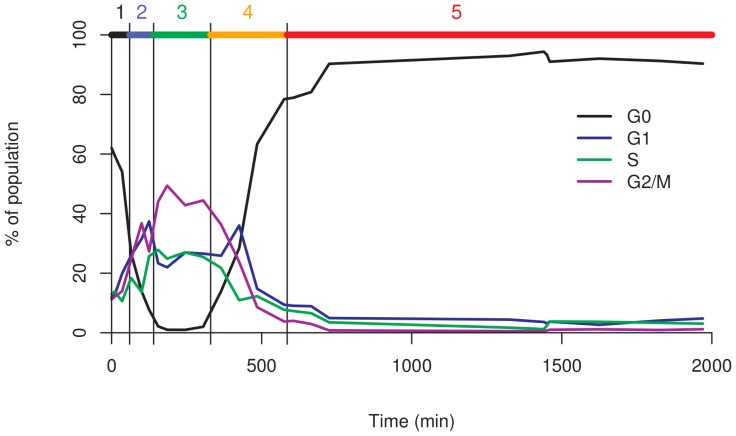
Time courses of the relative proportions of the cell populations during the various cell cycle stages, using SYTOX® Green fluorochrome (G0: black; G1: purple; S: green and G2/M: magenta). Colored scale indicates the stages as defined in Figure 2.

To identify the different cell growth stages, we analyzed culture growth kinetics by following cell density during a single culture in standard YEPD medium (Figure 1). Samples were collected at different times, and classified according to five growth steps as defined from the growth curve: the lag phase from inoculation to 35 min, the start of growth from 65 min to 125 min, the exponential stage from 155 min to 305 min, the slowdown phase from 365 min to 575 min, and the last phase during which the cells reached the G0 stage corresponding to a quiescent status (Figure 2). The growth kinetics data facilitated comparisons of cell populations in similar growth stages. DNA staining with SYTOX Green allowed following the relative proportion of cell populations at the four different cell cycle steps, G1, S, G2/M and G0 (Figure 3), as further detailed in this paper.

### Changes in Fluorochrome Signal with Culture Growth

We determined the progression of the cytometric signal for each of the fluorochromes throughout the cell cycle. In particular we recorded the fluorescence pattern and the apparent cell size (the term “apparent” is used as the cells were fixed, and consequently the FSC signal probably represented a size significantly different to that of living cells).

**Figure 4:**
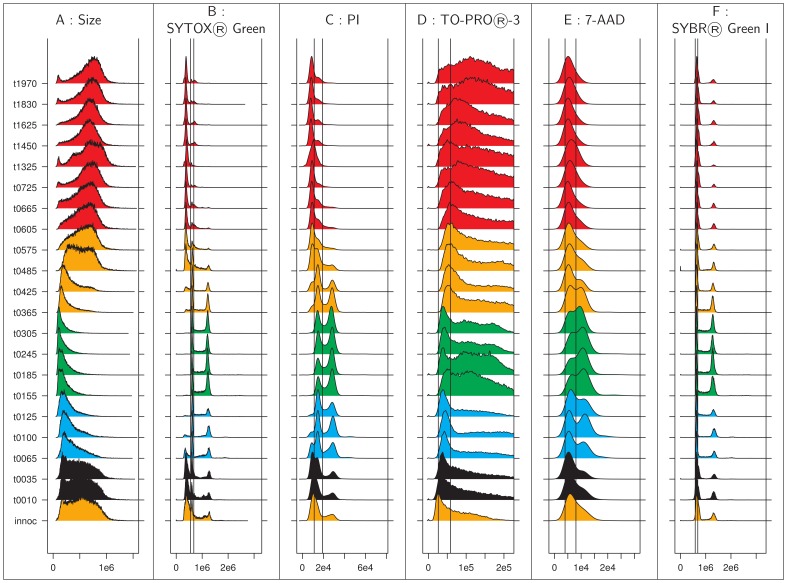
Evolution of the cytometric signal during cell culture (time in min) with A: cell size (Forward SCatter), B: SYTOX® Green, C: propidium iodide (PI), D: TO-PRO-3, E: 7-aminoactinomycin D (7-AAD) and F: SYBR® Green I. The x axes are in arbitrary units. The y axes correspond to relative frequencies adjusted for each signal. Each histogram represents about 20000 *cells* Colors correspond to the different growth stages as defined in Figure 2. For B to F, the vertical lines indicates the median signal 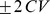 (coefficient of variation) corresponding strictly to yeast cells at G1 stage during the exponential phase.

The apparent cell size was small during the exponential phase and increased thereafter (Figure 4A).

The resolution and the pattern of fluorescent signal differed greatly according to both the fluorochrome used and progression through the cell cycle. The signal obtained with TO-PRO-3 (Figure 4D) showed very wide peaks and low intensity, and was unrelated to cell DNA content, rendering it useless for our purpose. The signal with 7-AAD (Figure 4E) was difficult to resolve as wide peaks masked the S phase. The widely used fluorochrome PI allowed satisfactory signal resolution (Figure 4C) and intensity, but only SYTOX Green (Figure 4B) and SYBR Green I (Figure 4F) achieved very highly resolutive signals. Two behaviors could be distinguished, with patterns either evidencing or not a signal for a sub-G1 peak. For identical cell population SYTOX Green or PI labellings presented a signal for samples not in the exponential stage, that was weaker than that of the G1 signal during the exponential phase. In contrast, no sub-signal population was detectable when 7-AAD or SYBR Green I was used for labeling. As cells originated from the same fixed populations, it can be concluded that the sub-signal peak does not correspond to the presence of small amounts of DNA, *i.e.* a “sub-G1” signal. Moreover, at this stage, not noticeable dead cell population was detected. All these data indicated that this “sub-G1” signal corresponds in fact to the signal of cells in G0 state. Thus, SYTOX Green allowed us to clearly distinguish between four cell cycle stages G1, S, G2/M and G0 in one-step labeling.

### Fluorochrome Signal Intensities

**Figure 5:**
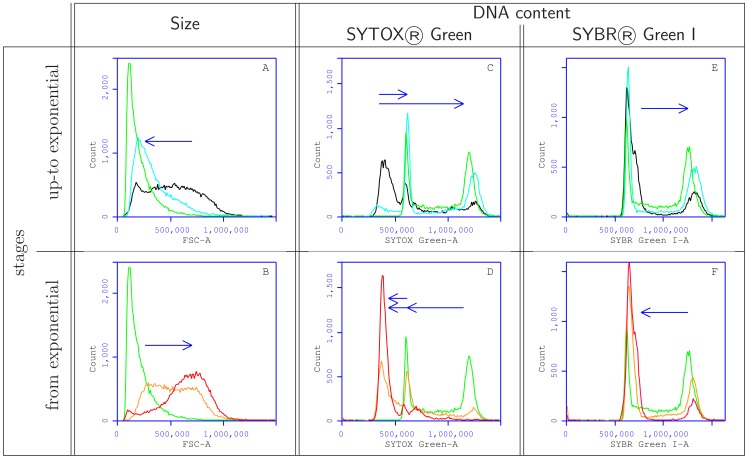
One dimensional representation of the apparent cell size (A, B), and DNA content as revealed by SYTOX Green (C, D) and SYBR Green I (E, F) labeling. Arrows underline the main evolutions of the DNA content from the lag phase to exponential growth (A, C, E) and from exponential growth to the stationary phase (B, D, F). Colors correspond to the different stages as defined in Figure 2.

Analysis of the fluorescence signals (median and coefficient of variation [CVCVcoefficient of variation] of the mean value of the DNA content, Table 1) obtained with cells in the exponential phase (G1 and G2/M) or stationary phase (G0) were represented in one dimension with adapted gatings. This facilitated comparison of standard cytometric parameters for the different labelings. From these data, and as illustrated above, labeling with SYBR Green I or SYTOX Green resulted in high intensity signals, with less than 5% CV and a 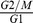 ratio of almost two. The signal with PI was satisfactory, but the CV was higher and the 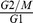 ratio significantly lower. The 7-AAD labeling signal was clearly informative and that of TO-PRO-3 was not usable. A sub-G1 peak was only observed with SYTOX Green and PI staining (Figure 4 and 5). In these last two cases, the G0 signal was 40% weaker than the G1 signal (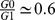).

**Table 1:** Signal intensity with different fluorochromes during the exponential cell growth phase (G1 and G2/M) or stationary phase (G0).

Fluorochrome	Specificity	G1		G2/M		G0		Ratio	
		median	CV	median	CV	median	CV	G2/G1	G0/G1
SYTOX® Green	dsDNA minor groove	616127	0.05	1213310	0.04	386217	0.12	1.97	0.63 *y*
PI	DNA, RNA	15234	0.13	28172	0.08	9083	0.20	1.85	0.60 *y*
TO-PRO®-3	dsDNA	42316	0.19	128121	0.23	109336	0.46	3.03	2.58 *n* *
7-AAD	DNA (CG-selective)	6009	0.17	10472	0.13	5628	0.26	1.74	0.94 *n*
SYBR® Green I	DNA, RNA	633999	0.04	1280899	0.04	665604	0.07	2.02	1.05 *n*

Global signal of low quality and upper G0 fluorescence.

^y^ presence of a sub-G1 peak out of the exponential growth.

^n^ absence of a sub-G1 peak out of the exponential growth.

### Consequences of the Stage of the Cell Cycle on Apparent Cell Size and DNA Fluorescence Signal

A more detailed analysis using the most resolutive fluorochromes evidencing or not the sub-G1 signal, viz. SYTOX Green and SYBR Green I, was used to evaluate the impact of the stage of the cell cycle stage on the apparent cell size and on DNA fluorescence signals (Figure 5A and B).

The size distribution observed was narrower and the apparent size itself was about six-fold lower for cells in exponential stage than for cells in stationary phase (G0). With SYTOX Green, the peak associated with G1 was narrow, mostly well separated and had a well detectable “sub-G1” signal which however did not correspond to a sub-G1 population in apoptosis (Figure 5C and D). In contrast, with SYBR Green I, the G0/G1 peak was wider for cells not in exponential growth, but its intensity remained similar to that of G1 (Figure 5E and F). In these patterns, it was also possible to visualize a cycling population during stationary phase, mostly revealed by the presence of a narrow, small G1 peak (red curve on Figure 5B and D). This may reflect continuing yeast growth after the diauxic shift.

Thus, a more accurate analysis of the cell cycle was obtained by combining DNA fluorescence signals and cell size in a two-dimensional representation.

### Identification of Cells in G0 with Single DNA Labeling

**Figure 6:**
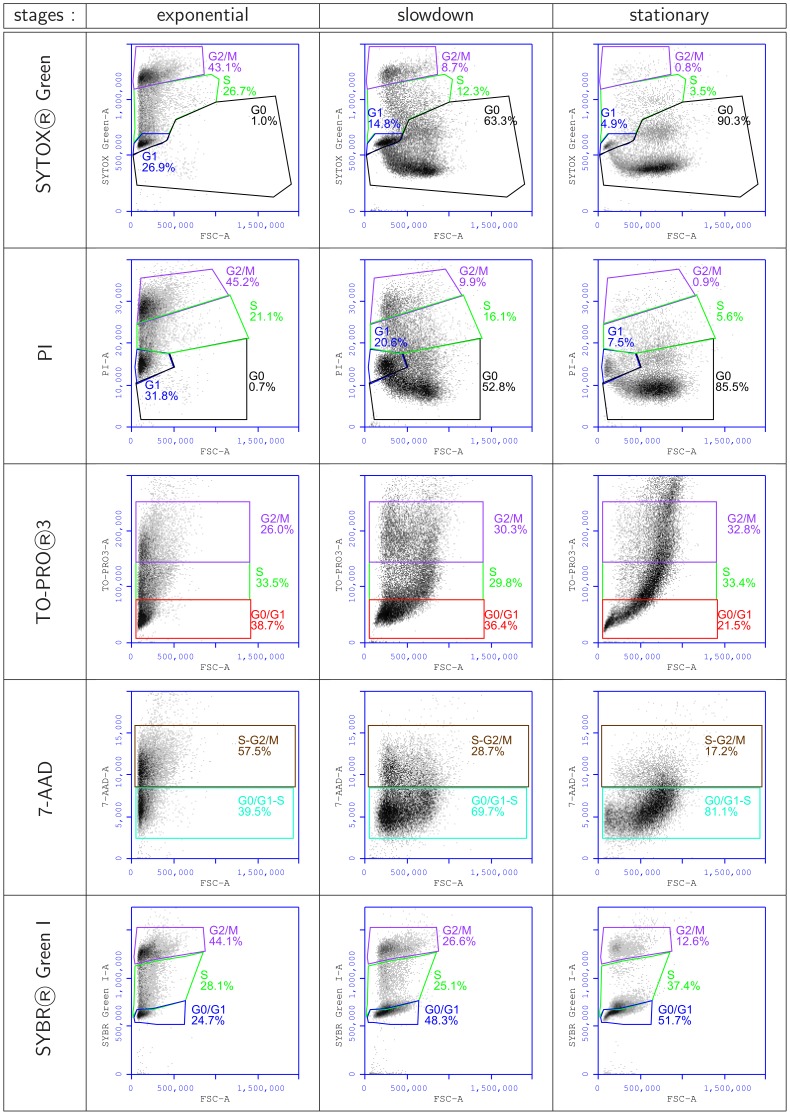
Two dimensional representation (DNA staining signal/apparent size) used to determine gating parameters with different fluorochromes (SYTOX® Green, PI, TO-PRO®-3, 7-AAD and SYBR® Green I). Gating was optimized for the whole growth experiment, and is summarized by the representation of yeast populations at three typical stages: exponential (245 *min*), slowdown (485 *min*) and stationary (725 *min*

Analysis of DNA fluorescence signals according to cell size allowed the determination of gating to characterize the different cell cycle steps (Figure 6). The gatings were designed for cells in exponential static growth phase for all data, and optimized for the entire tracking whenever possible. These gatings are to some degree arbitrary and clearly incorrect for cells in stationary and even slow down phases. They are given only to help with the comparison of the different fluorochromes. Cell cycle analysis was only done with SYTOX Green gating (Figures 3 and S1). For fluorochromes with no (TO-PRO3) or low (7-AAD) resolution in one dimension, no improvement was observed with these two dimensional representations. Also, with highly resolutive labeling (SYBR Green I), there was no improvement and the results seemed to indicate that the cells were continually cycling. In contrast, with PI and especially SYTOX Green, which revealed a true sub-G1 cell population, the two dimensional representation allowed clear visualization of cells which both contained less DNA and were larger than other cells. This pattern was observed as growth slowed down with the yeast culture almost in the stationary phase, characterizing the G0 stage. Thus, labeling with SYTOX Green or PI allowed a clear and straightforward discrimination between G0 and G1 cells. With these two fluorochromes, fixed gating parameters allowed the identification of four of the five cell cycle steps, whereas labeling with SYBR Green I was clearly unsatisfactory, due to a poor discrimination between cells at stages S and G0.

### Analysis of the Rate of Cell Entry into G0

To analyze the rate of cell entry into G0, cells were prepared as described in M&M with resuspension in water before SYTOX Green labeling. The evolution of the repartition between the different cell cycle steps (Figure S1) indicates that cells in S phase reached G2/M stage, with the corresponding population increasing directly after cell transfer to water. The population of G0 cells appeared clearly at about 100 *min*.

## Discussion

We investigated standard yeast culture growth to define the typical cell cycle stages through which the cells progress. This allowed us to study cells at characterized physiological stages. We then evaluated different sample preparation protocols and several DNA-binding dyes to assess the cell cycle in *S. cerevisiae* by FCM through DNA content evaluation. Various combinations of cell washing, overnight incubation and the presence of the detergent Nonidet-40 (Sigma) were tested but did not improve the outcome (data not shown). However, analysis of the results revealed that there may be misinterpretations during such analyses of *S. cerevisiae* cultures. Indeed, depending on the fluorochrome used, two different types of labeling behavior could be observed: those resulting in the expected, but not separated, G0 and G1 peaks and those with which G0 cells with a clearly lower DNA content were apparently detected. However, the narrowness of this peak was surprising for cells supposed to have fractional DNA content, whereas a larger population would have been expected. When G0 was detected, the fluorochrome signal of cells in G0 was underestimated by 40%; this result could be misinterpreted as indicating the existence of a “sub-G1” population. We did not take the diauxic shift into account because few cells were at this stage, although this small population was identified as being in G1 phase during cell cycle analysis, in particular when using SYTOX Green (Figure 6).

Attempts to induce cell apoptosis (with acetic acid) and thereby identify the characteristics of a true sub-G1 peak were unsuccessful (data not shown). This could be due to the strain used in this study (EC1118), as the response to acetic acid may greatly vary between yeast strains [5]. Several published papers seemed to wrongly interpret the G0 peak as a “sub-G1” population, although the corresponding cells were probably not in apoptosis [6], [7]. This misinterpretation may even have lead to erroneous discussions, and in particular, suggestions of the existence of pathways to explain the discrepancies in the results between the detection of “sub-G1” populations and TUNEL analyses. Our study shows that a number of conclusions may need to be reassessed.

We validated DNA-binding dyes for assessing the cell cycle in *S. cerevisiae*: SYTOX® Green which achieved good resolution, and PI with a lesser efficiency. As previously reported [8] SYTOX® Green exhibited a stable signal with only small variations (chronological, fluorescence or cell quantum) due to either yeast or fluorochrome, and the signals were more accurate than those of PI. In the tests we performed, PicoGreen, structurally very similar to SYBR Green I, resulted in a fluorescent signal intensity similar to that of SYTOX® Green, but with a lower signal for G0 cells (data not shown). Differences in the staining ability of fluorochromes observed in the present study were likely due to site binding and size characteristics of each dye, but unfortunately we were unable to access this information for further interpretation.

Two factors may affect fluorochrome-binding to DNA, resulting in a lower fluorescent signal for cells in G0, in particular when using SYTOX® Green and PI: either DNA compaction or changes in the cell wall thickness, composition and organization. Further investigations should be performed to discriminate between these two factors.

Cell cycle analysis may also be used to evaluate cell population in apoptosis (characterized by a sub-G1 population) which could be determined with SYBR Green I fluorochrome, allowing a discrimination between cycling and dying cells through true DNA content determination. This DNA content estimation can also be used to determine DNA ploidy. For this type of application, it is particularly important to use appropriate reference samples for different species.

Several fluorochromes are available for estimating cellular DNA content by FCM. However, careful selection of the most suitable reagent is required for optimal accuracy in the assessment of cell cycle distribution with *Saccharomyces cerevisiae* cells. In conclusion we can recommend SYTOX Green for cell cycle analysis, as it allowed one-step discrimination between G0, G1, S and G2/M stages, and SYBR® Green I when accurate determination of cell DNA content is required, as for sub-G1 identification or DNA ploidy application.

## Materials and Methods

### Yeast Strain and Growth Cell Culture

Experiments were performed with the diploid *S. cerevisiae* strain BY4743 (EUROSCARF, [9]). Standard Yeast Extract Peptone Dextrose medium (YEPDYEPDYeast Extract Peptone Dextrose medium) was used for all cultures. For a short growing time, a 16 *h* culture was used to inoculate the medium to give an 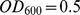. Aliquots of 10 *ml* of this inoculated medium were distributed into a series of double-cap 13 *ml* tubes tightly fixed on a Vibramax 100 (Heidolph, Schwabach) 3 *mm*-vibration orbital shaker platform set at 450 *rpm* and incubated at 28°*C*. One tube was used at each time point, for OD measurement, and cytometric analysis.

### Growth Stages

To characterize the different cell growth phases, cultures were diluted (from 1 to 10 fold) in water. OD was measured on a Nanodrop ND-1000 Spectrophotometer (Thermo Scientific) at wavelengths from 550 to 700 *mm*. The means of at least three measurements from 586 to 616 *mm* were calculated and are reported to minimize low measure variations. Sample aliquots were quickly fixed for cytometry analysis, at various times from inoculation to 33 *hours* later. Growth phases were defined as time after inoculation by the linearity of the OD log signal. Parameters were adjusted by a Gompertz function using R statistical software version 3.0.1 and the package “cellGrowth” [10], [11].

### Cell Preparation

Cell culture aliquots (about 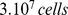, 5 *ml* to 300 µ*l*) were centrifuged. The cells were resuspended in 500 µ*l* of water and fixed in 10 *ml* of 75% ethanol, added dropwise under continuous vortexing to avoid cell agglomeration. Samples were stored at least one night at 4°*C*. The samples were then centrifuged for 5 *min* at 1962 *g* and cell pellets resuspended in 1 *ml* PBS and centrifuged for 1 *min* at 13000 *g* at room temperature. The supernatant was discarded and enzymatic treatments were then performed to eliminate RNAs and proteins: the pellet was resuspended in 500 µ*l* RNAse A (2 *mg*.*ml* ^−1^ in 10 *mmol.l* ^−1^ Tris-Cl and 15 *mmol.l* ^−1^ NaCl), incubated for 1 *h* at 37°*C*, and centrifuged; the pellet was resuspended in 200 µ*l* proteinase K (1 *mg.ml* ^−1^ in PBS), sonicated for 15 *s* in an ultrasonic bath (Branson Sonifier), incubated for 1 *h* at 50°*C* and centrifuged. The pellet was resuspended in 500 µ*l* PBS, sonicated for 2 *min* and stored on ice until analysis.

To analyze the rate of cell entry into G0, cultures were harvested during the exponential growth phase and aliquots of about 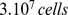 were pelleted by centrifugation at 4415 *g* for 1 *min*. The supernatant was removed (using a vacuum pump) to eliminate nutrients, and the cells were resuspended in 600 µl H2O, and incubated up to 4 *h* before fixation and labeling with SYTOX® Green for cell cycle analysis.

### Cytometry

The performance of the BD Accuri C6™ flow cytometer was validated using 6- and 8- peak fluorescent bead mixtures provided by the manufacturer, and according to instructions (Becton, Dickinson and Company, Franklin Lakes). Analysis was based on light-scatter and fluorescence signals produced from 20 *mW* laser illumination at 488 *nm*. Signals corresponding to forward angle- and 90°-side scatter (FSC and SSC) and fluorescence were accumulated, the fluorescence signals (pulse area measurements) being screened by the following filter configurations: (a) FL1: a 530/30 *nm* band-pass filter for SYTOX® Green and SYBR Green I fluorescent, (b) FL2: a 585/40 *nm* band-pass filter (not used in this study), and (c) FL3: a 670 *nm* long-pass filter for 7-AAD and PI fluorescences and a 14.7 *mW* laser illumination at 640 *nm* enabling fluorescence through (d) FL4: a 675/25 *nm* band-pass filter for TO-PRO-3 signal. Threshold level was set at 40,000 for FSC. For the identification of the region corresponding to yeast cells, data were collected to a total count of 20,000 *cells*. The flow cytometer was routinely operated at the Slow Flow Rate setting (14 µ*Lsample/min*) and yeast samples presented more than 95% singlets (on FSC-A/FSC-H representation).

Cells were labeled by dilution of 25 µ*l* aliquots of cells (1.25×10^6^ *cells*) in 200 µ*l* of fluorochrome-containing PBS to reach the final concentration indicated below and 1000 *eps* with a slow flow rate (14 µ*l.min^−1^*). The concentrations used were based on the quantities of fluorochrome per cell described in the literature data [12], [13]; we verified that a stable signal was obtained, at least within a two-fold variation of the concentration. Labeled cells were kept at least 30 *min* in the dark at room temperature and signal stability was verified by reanalyzing the initial samples at the end of the full run. In the work reported here, we compared five current fluorescent DNA dyes: SYTOX® Green (1 µ*mol* ^−1^), TO-PRO-3 (1 µ*mol* ^−1^, overnight labeling), PI (2.2 µ*g.ml*), 7-AAD (10 µ*g.ml* and SYBR Green I (1/2250). Samples were analyzed directly in the labeling buffer with the C6 cytometer.

### Cytometry Data and Graphic Representation

Cytometry data files are available in the Cytobank database at the following addresses: https://www.cytobank.org/cytobank/experiments/25061 and https://www.cytobank.org/cytobank/experiments/25066.

Signal time courses were drawn using flow cytometry packages of the Bioconductor project running with R [10], [14]–[17]. The scale of the fluorochrome signal was set so that the G1 intensity peak pointed to the 200/1000 of the whole x scale. Population gates are optimized for static gating across the cell cycle. This induced some wrong gatings at the end of the culture, especially for G0 populations detected with SYBR® Green I labeling (Figure 6) : in these cases, some cells were counted as being in S-phase.

## Supporting Information

Figure S1 **Analysis of the evolution of the different cell populations after resuspension of yeast cells in water.** Exponentially growing yeast cells were incubated in pure water before analysis of cell cycle stages using SYTOX® Green (G0: black; G1: purple; S: green and G2/M: magenta).
(TIF)
