# Supplementary figures and images for: A Simple FCM Method to Avoid Misinterpretation in Saccharomyces cerevisiae Cell Cycle Assessment between G0 and Sub-G1
Source: PLoS One. 2014 Jan 2;9(1):e84645. doi: 10.1371/journal.pone.0084645 (PMC3879310; doi:10.1371/journal.pone.0084645)

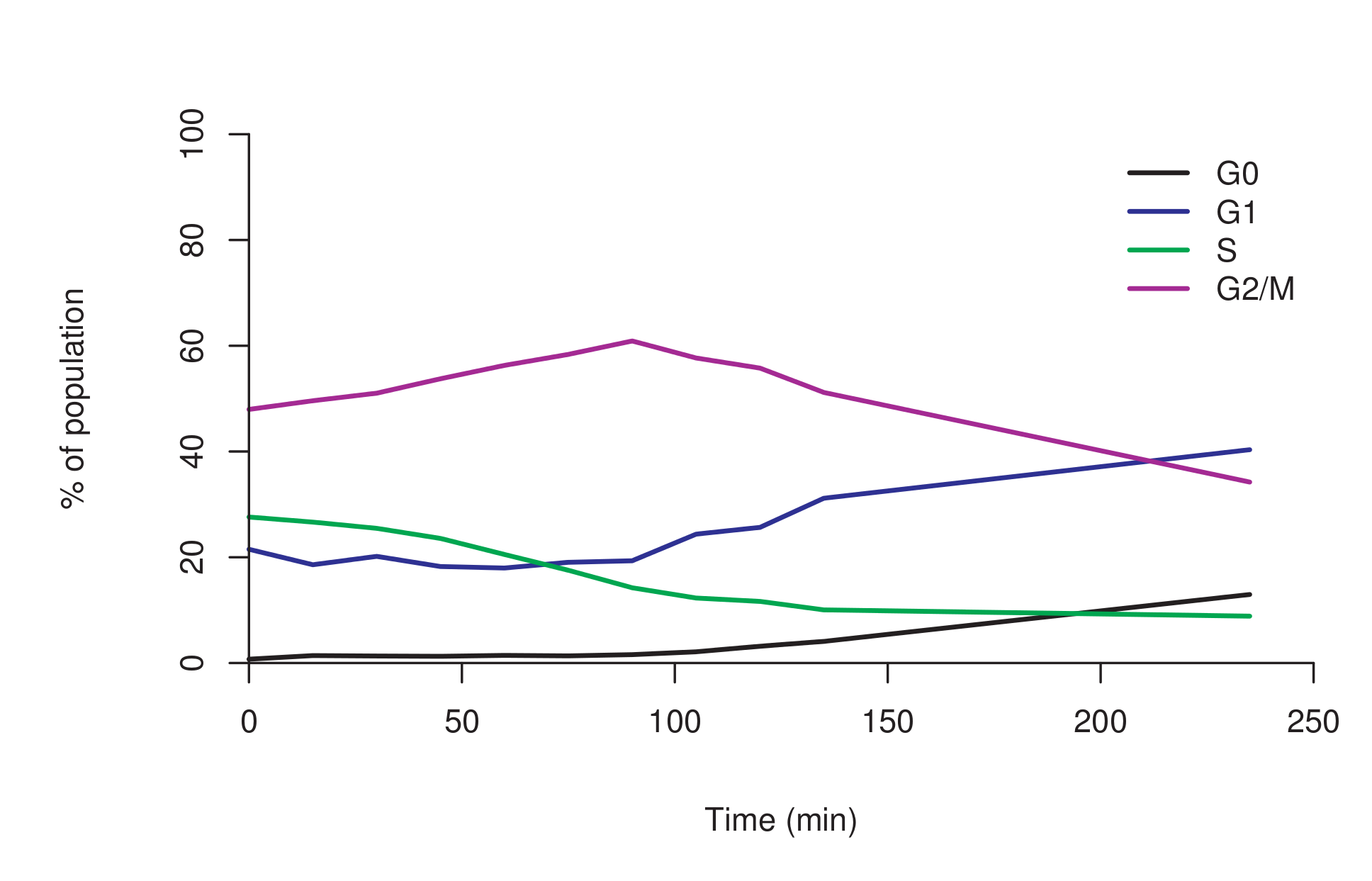

Supplement: Figure S1 — Analysis of the evolution of the different cell populations after resuspension of yeast cells in water. Exponentially growing yeast cells were incubated in pure water before analysis of cell cycle stages using SYTOX® Green (G0: black; G1: purple; S: green and G2/M: magenta). (TIF) [file pone.0084645.s001.tif]
